# 3D NiCoW Metallic Compound Nano-Network Structure Catalytic Material for Urea Oxidation

**DOI:** 10.3390/nano14221793

**Published:** 2024-11-07

**Authors:** Zuoyuan Liang, Lang Yao, Yipeng Zhang, Sirong Li, Xuechun Xiao

**Affiliations:** School of Materials and Energy, Yunnan University, Kunming 650091, China; lzy518032@163.com (Z.L.); yl6626167@163.com (L.Y.); z2947207693@163.com (Y.Z.); srlili@ynu.edu.cn (S.L.)

**Keywords:** NiCoW catalyst, urea oxidation reaction, oxygen evolution reaction, electronically controlled

## Abstract

Urea shows promise as an alternative substrate to water oxidation in electrolyzers, and replacing OER with the Urea Oxidation Reaction (UOR, theoretical potential of 0.37 V vs. RHE) can significantly increase hydrogen production efficiency. Additionally, the decomposition of urea can help reduce environmental pollution. This paper improves the inherent activity of catalytic materials through morphology and electronic modulation by incorporating tungsten (W), which accelerates electron transfer, enhances the electronic structure of neighboring atoms to create a synergistic effect, and regulates the adsorption process of active sites and intermediates. NiCoW catalytic materials with an ultra-thin nanosheet structure were prepared using an ultrasonic-assisted NaBH_4_ reduction method. The results show that during the OER process, NiCoW catalytic materials have a potential of only 1.53 V at a current density of 10 mA/cm^2^, while the UOR process under the same conditions requires a lower potential of 1.31 V, demonstrating superior catalytic performance. In a mixed electrolyte of 1 M KOH and 0.5 M urea, overall water splitting also shows excellent performance. Therefore, the designed NiCoW electrocatalyst, with its high catalytic activity, provides valuable insights for enhancing the efficiency of water electrolysis for hydrogen production and holds practical research significance.

## 1. Introduction

With the gradual development of society, both industry and agriculture have made significant progress. However, this progress has been accompanied by energy shortages and environmental degradation, which are constraining the advocated strategies for sustainable development. Hydrogen, as an ideal sustainable energy source, possesses characteristics such as being green and pollution-free, and having high energy density and wide applicability. Among many new energy sources, it has a higher availability and can replace traditional fossil fuels to reduce environmental pollution [1]. Water electrolysis is a primary method for hydrogen production. Water electrolysis consists of the oxygen evolution reaction (OER) at the anode and the hydrogen evolution reaction (HER) at the cathode [2]. The OER involves a four-electron transfer process [3,4], with a theoretical potential of 1.23 V vs. RHE. Due to its high reaction energy barrier and slow reaction kinetics, these factors significantly limit the development of water electrolysis for hydrogen production. Therefore, improving the efficiency of the anode oxidation reaction is a current research focus [5]. The urea oxidation reaction (UOR) occurs at the anode during the electrolysis of urea-containing solutions. When UOR replaces OER, its theoretical voltage is only 0.37 V vs. RHE [6,7]. Additionally, industrial wastewater and human and animal urine are rich in urea, which has both polluting and resource characteristics [8,9]. Improper disposal of this urea can cause serious pollution, as the ammonia, nitrites, nitrates, and nitrogen oxides produced from urea can contaminate air quality and groundwater, indirectly affecting human health and safety [10,11]. Moreover, urea is a hydrogen storage chemical with a hydrogen content of up to 6.71 wt%, providing a rich source of hydrogen. However, UOR also has areas that need improvement, as the reaction involves a six-electron transfer process (CO(NH_2_)_2_ + 6OH^−^ → N_2_ + CO_2_ + 5H_2_O + 6e^−^). Its thermodynamics and kinetics are relatively slow, thus necessitating the design of catalysts to enhance its electrochemical performance [12,13,14,15].

High-performance, low-cost UOR catalysts are the most ideal electrocatalysts. Initially, precious metals such as Pt, Pd, and Ag were used to catalyze the UOR process. As shown in Table 1, the performance parameters of common noble metals in electrocatalysis are presented. However, their high cost and limited availability significantly restricted their use [16]. Consequently, the development of bimetallic or multi-metallic catalysts has become a research hotspot. Among these, relatively inexpensive nickel-based electrocatalysts have been developed in alkaline media, showing higher current densities and lower overpotentials [17,18]. Boggs et al. [19] discovered that the onset potential of the UOR reaction is the same as the potential for the oxidation of Ni(OH) to NiOOH, indicating that nickel-based materials are key active sites for the UOR reaction. Hainan Sun et al. [20] used a cation exchange strategy to synthesize Ni-doped CuO nanowire arrays grown on 3D Cu foam. This demonstrates that Ni, as a driving force center, can effectively enhance urea adsorption and stabilize reaction intermediates, resulting in high-performance urea oxidation catalysts. Shi-Zhang Qiao et al. [21] developed an oxygen-anion-engineered nickel catalyst to suppress the competitive oxygen evolution reaction during the urea oxidation process, achieving an ultra-high current density of 323.4 mA cm^−2^ at 1.65 V. Although Ni-based catalytic materials have made some progress, their performance in terms of overpotential and stability in the overall urea decomposition process still lags behind noble metals such as Pt and Ru. Therefore, combining Ni-based catalytic materials with other transition metals to form bimetallic or polymetallic catalysts could exhibit superior electrocatalytic performance [22,23]. Building on Ni-based catalysts, incorporating other metal elements can enable electronic modulation to enhance catalyst activity, increase the electrochemical active surface area, and create more reactive sites. This can improve the reaction rate and efficiency, thus promoting the reaction [24,25]. In addition, this study doped W elements into NiCo, which affected the electronic structures of Ni and Co due to local environmental changes. This resulted in an increased concentration of oxygen vacancies, leading to a surface rich in electrocatalytic active sites [26]. Xinqiang Wang et al. synthesized a multilayer and porous W-doped CoP nanosheet array on carbon cloth via liquid-phase reaction and phosphidation processes. The exceptional electrocatalytic performance of the resulting material is primarily attributed to the porous W-doped nanosheet array, which not only provides abundant exposed active sites but also accelerates electrolyte infiltration and bubble diffusion, thereby effectively enhancing the electrocatalytic performance [27].

In this study, a three-metal NiCoW catalyst with an ultrathin nanosheet structure was successfully prepared using a reduction method with ultrasound assistance. According to the final experimental results, Ni, as an electron donor, possesses a high oxidation state and is the main catalytic site driving rapid reactions. Through morphological and electronic modulation as well as inter-element synergy, the electrochemical active surface area and interfacial charge transfer capability of the NiCoW catalyst are increased, promoting the formation of *OOH and enhancing its inherent activity, thereby improving the OER performance [28]. Electrochemical tests using a rotating disk electrode system showed that after the addition of urea, the NiCoW catalyst achieved a current density of 10 mA/cm^2^ for UOR at an overpotential of only 1.31 V, which is 220 mV lower compared to OER. Stability is a crucial performance metric for electrocatalysts. The prepared NiCoW electrocatalyst was subjected to 3000 cycles of cyclic voltammetry (CV) testing. After this, linear sweep voltammetry (LSV) was performed on the NiCoW catalyst at a current density of 10 mA/cm^2^. The potential after 3000 cycles of CV remained at 1.34 V, consistent with the potential before the CV tests, indicating that the catalyst exhibits good stability. The urea-assisted overall water splitting also shows excellent performance, with no significant change in current density after 25 h of testing. It can be seen that the NiCoW catalyst exhibits good electrochemical performance. The Ni-based metal electrocatalyst designed in this work could be a candidate for non-precious metal catalysts.

## 2. Experimental

### 2.1. Materials

Cobalt(II) nitrate hexahydrate (Co(NO_3_)_2_·6H_2_O), nickel(II) nitrate hexahydrate (Ni(NO_3_)_2_·6H_2_O), sodium tungstate dihydrate (Na_2_WO_4_·2H_2_O), sodium borohydride (NaBH_4_), anhydrous ethanol (C_2_H_5_OH), and isopropanol (C_3_H_8_O) were purchased from Shanghai Macklin Biochemical, China, Co., Ltd. Shanghai, China. Nafion solution (5%) and acetylene black were used. The deionized water (18.2 MΩ cm) used in the experiments was purified using a ZYPFT-2-20T system from Sichuan Excellence Water Treatment Equipment Co., Ltd. Chengdu, China. All products were used as received without further purification.

### 2.2. Preparation of NiCoW and NiCo Catalyst

The preparation method used in this experiment was the NaBH_4_ reduction method. First, add 1 mmol of Ni(NO_3_)_2_·6H_2_O and 2 mmol of Co(NO_3_)_2_·6H_2_O into 15 mL of deionized water and 15 mL of absolute ethanol. Stir the mixture with a magnetic stirrer for 30 min until completely dissolved, referred to as Solution A.

**Figure 1:**
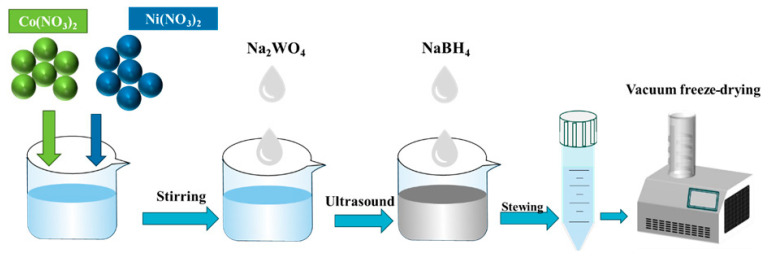
The preparation process of NiCoW.

Prepare Solution B by dissolving 1 mmol of NaWO_4_· 2H_2_O in 30 mL of deionized water and stirring for 30 min. Gradually add Solution B to Solution A, resulting in the formation of a floccule. Finally, under ultrasonic conditions, add 30 mL of 0.4 mol/L NaBH_4_ to the mixture until no bubbles are produced. After stirring for 2 h, five cycles were washed by centrifugation at 6000 rpm. The supernatant was removed, and the wet sample was vacuum freeze-dried to obtain the NiCoW catalytic material. The preparation process of NiCoW is shown in Figure 1.

As a control experiment, the NiCo catalyst was prepared under the same experimental conditions as described above, except that NaWO_4_·2H_2_O was not added. The amounts of Co(NO_3_)_2_·6H_2_O used were 1 mmol and 2 mmol, named NiCo-1 and NiCo-2, respectively.

### 2.3. Material Characterization

Phase characterization of the synthesized samples was performed using a Rigaku D/MAX-3B X-ray diffractometer (Rigaku Holdings Corporation, Tokyo, Japan), with a scanning range of 10° to 90°. The surface morphology and elemental distribution were analyzed using a Nova Nanosem 450 field emission scanning electron microscope (FEI, Hillsboro, OR, USA)from FEI. Morphological and selected area electron diffraction (SAED) analyses were conducted using a JEM-2100 (UHR) transmission electron microscope from (Nippon Electron Co. LTD, Otsu, Japan). Elemental and valence state analyses of the materials were performed using a PHI 5500 X-ray photoelectron spectrometer from (PHI, Tokyo, Japan) with Al/Mg as the dual anode target material. The pore size distribution and BET-specific surface area of the samples were determined using a NOVA 2200e gas adsorption analyzer. Since NiCo-2 exhibits superior overall performance in UOR compared to NiCo-1, subsequent characterizations will be based on NiCo-2.

### 2.4. Electrode Preparation and Electrochemical Measurements

All electrochemical data for this experiment were tested using a CHI760E electrochemical workstation (CHI, Beijing, China), which integrates a three-electrode system with a Compact Pine Rotator (Pine, San Jose, CA, USA). Before performance testing, the working electrode was first polished with polishing powder on a deer skin and then cleaned with deionized water to ensure that no surface contaminants interfered with the electron transfer during the test. The synthesized catalyst (7 mg) was mixed with acetylene black (3 mg), deionized water (490 µL), isopropanol (490 µL), and Nafion (20 µL), and sonicated for 30 min to prepare a catalyst ink. Then, 20 µL of the uniformly dispersed ink was applied in two separate deposits onto the rotating electrode surface to form a more uniform film, which was then dried at room temperature for 12 h. Before testing, high-purity nitrogen was bubbled through the electrolyte for half an hour to remove oxygen from the electrolyte (1 M KOH), and this nitrogen flow was maintained throughout the testing process. RDE (5 mm) and a standard Hg/HgO electrode, along with a platinum wire, were used as the working electrode, reference electrode, and counter electrode, respectively. Linear sweep voltammetry (LSV) was performed with a scan rate of 5 mV/s and a rotation speed of 1600 rpm (90% automatic compensation). The overpotential (η) obtained from the LSV curve was calculated using Equation (1).

η = E_(RHE)_ − 1.23 (1)

The Tafel slope was determined by fitting the LSV results using Tafel Equation (2), where b and j represent the Tafel slope and current density, respectively.

E_(RHE)_ = a + b log|j| (2)

The electrochemical impedance spectroscopy (EIS) was recorded over a frequency range from 100 kHz to 0.1 Hz, with the experimental voltage as the initial voltage. Additional calculations include the following: determining the double-layer capacitance (C_dl_) in the voltage range of 0.2 to 0.3 V (vs. RHE) with scan rates ranging from 20 to 100 mV/s and establishing a linear relationship between scan rate and current density. The specific calculation formula is shown in Equation (3), where v is the scan rate of the cyclic voltammetry test, and ja and jc are the current densities at 0.25 V (vs. RHE) after fitting the cyclic voltammetry curve.

(3) $$Cdl=\frac{\left|ja-jc\right|}{2v}$$

To assess the stability of the NiCoW catalyst, stability tests were performed by comparing the LSV curves before and after 3000 cycles of CV testing. If the changes fall within a reasonable range, it indicates good stability of the catalyst. All potentials reported in this study were referenced to the reversible hydrogen electrode (RHE), and the Nernst equation in Equation (4) was used to calculate the potential of the RHE, where E(Hg/HgO) represents the standard potential of the mercury (Hg) and mercuric oxide (HgO) electrode. The term “0.059 × pH” indicates the effect of pH on the electrode potential, showing a linear relationship between acid–base strength and potential. The term “0.098” is a constant related to the standard hydrogen electrode.

E_(RHE)_ = E_(Hg/HgO)_ + 0.059 × PH + 0.098 (4)

## 3. Results and Discussion

### 3.1. Characterization of Morphology and Structure

**Figure 2:**
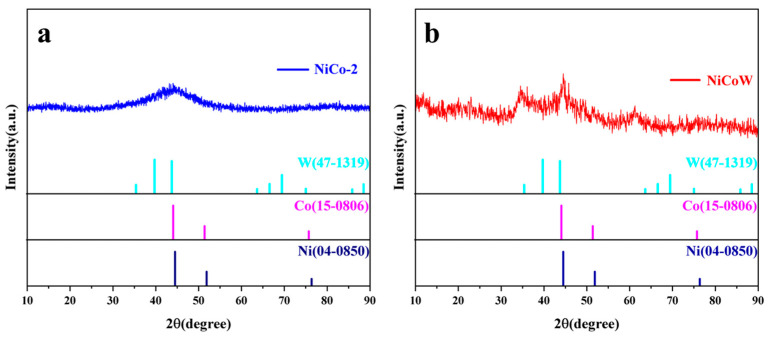
XRD patterns of (**a**) NiCo-2 catalyst and (**b**) NiCoW catalyst.

To determine the composition of the samples, X-ray diffraction (XRD) phase characterization was conducted on the NiCo-2 samples. The results, shown in Figure 2a, reveal characteristic peaks of Ni at 2θ = 44.5°, 51.8°, and 76.4°, which are consistent with the standard XRD pattern JCPDS No:04-0850 and correspond to the (111), (200), and (220) crystal planes of Ni, respectively. Additionally, a characteristic peak of Ni_2_O_2_(OH) was observed for Ni at 21.6°, which may be attributed to the formation of nickel hydroxide due to a vigorous reduction reaction between nickel and water. Characteristic peaks for Co were observed at 2θ = 44.2°, 51.5°, and 75.8°, which match the standard XRD pattern JCPDS No:15-0806 and correspond to the (111), (200), and (220) crystal planes of Co, indicating successful preparation of the NiCo-2 catalyst samples. X-ray diffraction analysis was also performed on NiCoW, as shown in Figure 2b. The analysis revealed not only the characteristic peaks of Ni and Co, which are similar to those of the NiCoW catalyst, but also characteristic peaks of W at 2θ = 35.5°, 43.8°, and 63.7°, corresponding to the standard XRD pattern JCPDS No:47-1319. No impurity diffraction peaks related to other elements were observed in the XRD pattern, indicating that the NiCoW catalyst prepared has high purity and that the experimental process was rigorous.

**Figure 3:**
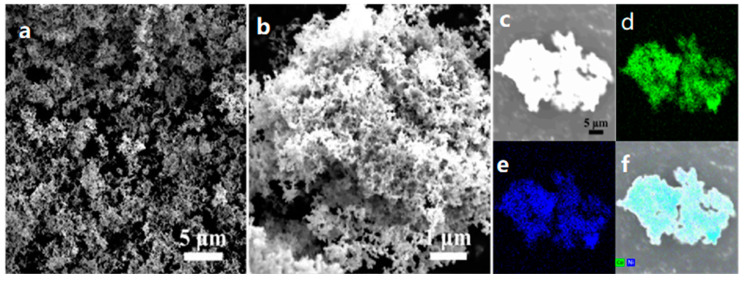
SEM images of NiCo-2 at (**a**) low and (**b**) high magnification. (**c**–**f**) Elemental mapping images of NiCo-2.

**Figure 4:**
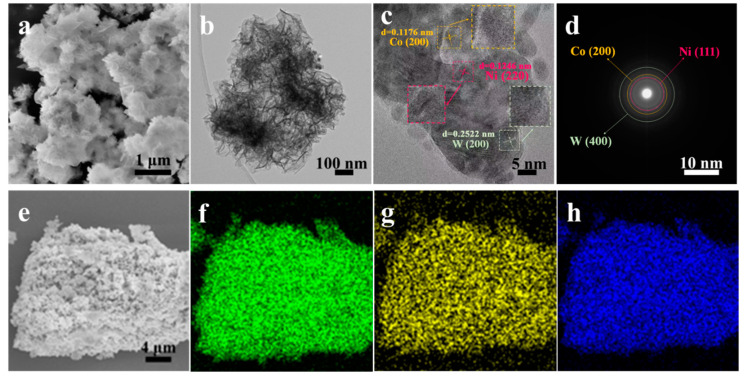
(**a**) SEM images of NiCoW, (**b**) TEM images of NiCoW, and (**c**) HRTEM images of porous NiCoW, (**d**) SAED pattern of NiCoW and (**e**–**h**) elemental mapping images of NiCoW.

The morphology and structure of the NiCo-2 and NiCoW catalyst materials were characterized using SEM and TEM. Figure 3a shows that the NiCo-2 catalyst material exhibits a stacked arrangement of particles with varying sizes, with some particles displaying slight agglomeration. As shown in Figure 3b, the particles are interconnected, forming numerous interlaced channels. As shown in Figure 4a, after adding sodium tungstate to form the NiCoW catalyst, the morphology of the catalyst material transforms from a particulate form to a three-dimensional ultrathin nanosheet structure. Some nanosheets agglomerate in certain regions to form flocculent structures. This transformation increases the specific surface area, significantly enhances electron transfer rates, and increases the number of active sites on the catalyst material, ultimately improving its catalytic activity [29,30]. The generation of gas bubbles during performance testing is a significant factor affecting the data results. The formed porous morphology of the material can provide more release channels for the generated hydrogen and oxygen during performance testing [31]. To draw more significant conclusions regarding the microstructure of the NiCoW catalyst, TEM characterization was performed on the NiCoW catalyst material. As shown in Figure 4b, the NiCoW sample exhibits a cotton-like flocculent structure, with some particles extending into ultrathin nanosheets, forming a three-dimensional morphology with a porous structure. The NiCoW catalyst exhibits a complex three-dimensional structure, consistent with the results described earlier from the scanning electron microscopy (SEM) characterization. In Figure 4c, the HRTEM image reveals lattice fringes in the NiCoW sample, indicating that the NiCoW catalyst is crystalline [32,33]. The 0.1176 nm lattice spacing can be attributed to the (200) plane of Co, the 0.1246 nm lattice spacing to the (220) plane of Ni, and the 0.2522 nm lattice spacing to the (200) plane of W, further confirming the formation of the NiCoW metallic compound. Figure 4d further shows that after Fourier transformation of the same region, diffraction rings corresponding to the elements Ni, Co, and W appear, also confirming the successful synthesis of the NiCoW catalyst. To more clearly illustrate the distribution of the three elements, element mapping images from Figure 3c–f and Figure 4e–h reveal that the elements in the NiCo-2 and NiCoW catalysts are evenly distributed across the scanned surfaces [34,35]. 

**Figure 5:**
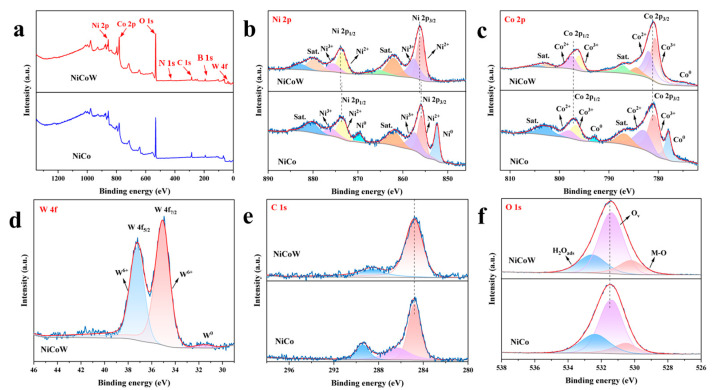
XPS spectrum of NiCoW and NiCo-2 catalysts: (**a**) full spectrum, (**b**) Ni 2p, (**c**) Co 2p, (**d**) W 4f, (**e**) C 1s, (**f**) O 1s.

To study the surface elemental valence states, X-ray photoelectron spectroscopy (XPS) was used to characterize the elemental chemical states and contents of the NiCoW and NiCo-2 catalysts. The full spectra shown in Figure 5a demonstrate that Ni, Co, W, C, and O elements are present in NiCoW, while Ni, Co, C, and O elements are present in NiCo-2. The presence of W only in NiCoW successfully confirms the preparation of the NiCoW catalyst. Additionally, the presence of B and N elements was discovered, possibly due to the solution reagents. The relative content of elements obtained from the full spectrum is shown in Table 2. The ratio of Ni and Co elements in the NiCoW material roughly conforms to a 1:2 ratio. In NiCo-2, the ratio of Ni to Co is relatively small, and the proportion of W elements in NiCoW is also small, which may be related to the volatilization caused by the temperature increase during the ultrasonic process. Figure 5b shows the Ni 2p XPS spectra for both samples, where the presence of Ni^3+^ and Ni^2+^ ions can be observed. The peaks at 855.9 eV and 873.8 eV correspond to Ni 2p_3/2_ and Ni 2p_1/2_, respectively, while the peaks at 861.7 eV and 880.2 eV are satellite peaks. The peaks at 852.3 eV and 869.7 eV correspond to Ni^0^, while the peaks at 855.8 eV and 873.42 eV correspond to Ni^2+^, and the peaks at 857.3 eV and 875.9 eV correspond to Ni^3+^. The remaining peaks are satellite peaks. Compared to NiCo-2, the Ni 2p_3/2_ and Ni 2p_1/2_ orbitals in NiCoW shift to slightly higher binding energies, indicating that the addition of W has an electronic modulation effect on the original sample [36,37]. Figure 5c shows the Co 2p XPS spectra. For the NiCo-2 sample, the peaks at 781.2 eV and 797.2 eV correspond to Co 2p_3/2_ and Co 2p_1/2_ orbitals, respectively. The peaks at 778.0 eV and 793.0 eV correspond to Co^0^, while the peaks at 780.8 eV and 797.1 eV correspond to Co^3+^, and the peaks at 782.9 eV and 801.3 eV correspond to Co^2+^. The remaining peaks are satellite peaks [38,39]. Similarly, the Co 2p_3/2_ and Co 2p_1/2_ peaks of the NiCoW sample shift slightly to lower binding energies. The peak at 781.0 eV corresponds to the Co 2p_3/2_ orbital, which shifts 0.2 eV to the right compared to NiCo-2. The peak at 796.3 eV corresponds to the Co 2p_1/2_ orbital, which shifts 0.9 eV to the right compared to NiCo-2. The peak at 774.5 eV corresponds to Co^0^. From the W 4f spectrum in Figure 5d, the peak at 35.1 eV corresponds to W 4f_7/2_, and the peak at 37.2 eV corresponds to W 4f_5/2_ [40,41]. The C 1s spectrum in Figure 5e shows that for NiCo-2, the peaks at 284.8 eV, 286.3 eV, and 289.4 eV correspond to C–C, C–O–C, and O–C=O bonds, respectively. In contrast, the NiCoW catalyst only shows C–C and O–C=O bonds. From the O 1s spectrum in Figure 5f, the peak at 530.2 eV is attributed to metal–oxygen bonds, the peak at 531.4 eV is attributed to oxygen vacancies, and the peak at 532.5 eV is attributed to adsorbed water.

**Figure 6:**
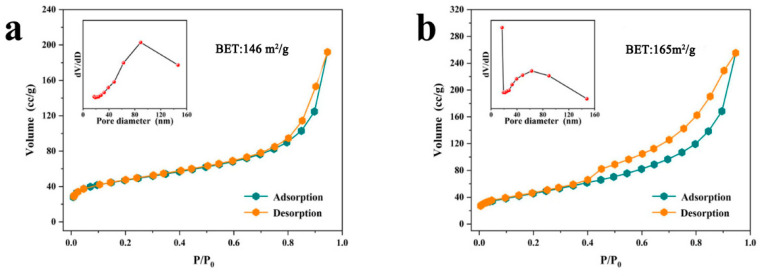
(**a**) N_2_ adsorption and desorption isotherm curve of the NiCo-2 catalyst, (**b**) N_2_ adsorption and desorption isotherm curve of the NiCoW catalyst.

The catalytic materials NiCo-2 and NiCoW were characterized using nitrogen adsorption/desorption analysis. Figure 6 shows type IV isotherms with H3 hysteresis loops for the catalytic materials NiCo-2 and NiCoW, indicating that both samples may have mesoporous structures. The specific surface areas of the samples were determined using the Brunauer–Emmett–Teller (BET) method, which were 146 m^2^/g and 165 m^2^/g, respectively. NiCoW has a larger specific surface area, which can expose more reactive sites and thus accelerate the catalytic reaction [42,43]. Table 3 lists the average pore diameters and pore volumes of the samples calculated using the Barrett–Joyner–Halenda (BJH) method. The average pore diameters were 89 nm and 17 nm, respectively. The average pore volumes were 0.249 cm^3^/g and 0.395 cm^3^/g, respectively. These data further confirm the presence of a mesoporous structure in the NiCoW catalyst material. The catalytic material NiCoW has a larger specific surface area, larger average pore volume, and smaller average pore diameter, which demonstrates that it creates a better catalytic environment and further accelerates the reaction.

### 3.2. OER Performance

**Figure 7:**
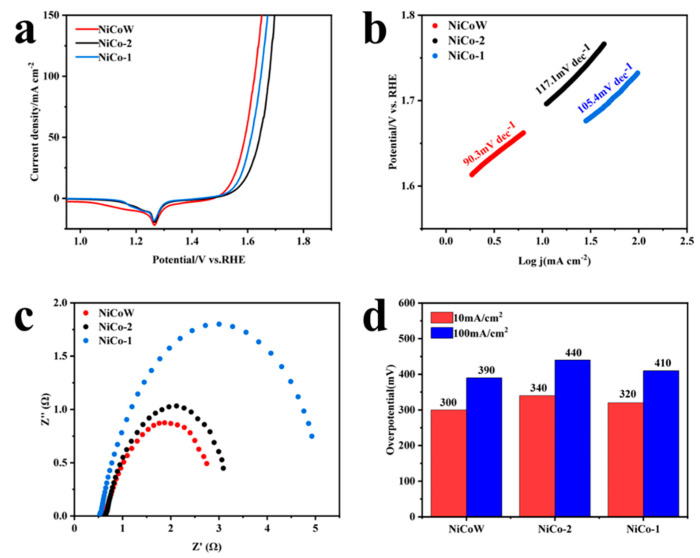
(**a**) Polarization curves of NiCo-1, NiCo-2, and NiCoW catalysts for 1 M KOH electrolyte. (**b**) Tafel slopes of NiCo-1, NiCo-2, and NiCoW catalysts. (**c**) Nyquist plots of NiCo-1, NiCo-2, and NiCoW catalysts. (**d**) The histogram of overpotentials at 10 and 100 mA cm^−2^.

**Figure 8:**
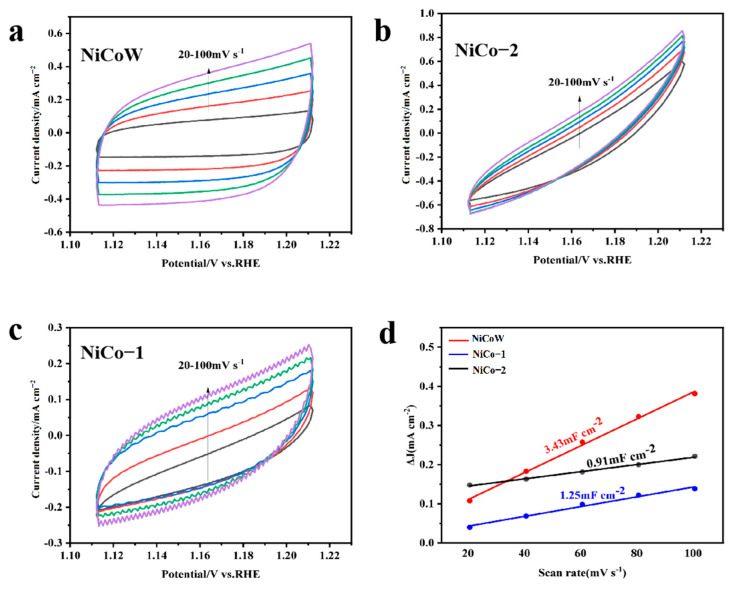
(**a**–**c**) Typical CV curves of 1.0 M KOH; scan rates range from 20 to 100 mV s^−1^. (**d**) C_dl_ of NiCoW, NiCo-1, and NiCo-2 catalysts. The different colors of the curves in the figure mean that we performed the CV cycles at different sweep speeds.

The OER performance of the samples was tested using a three-electrode system under alkaline conditions of 1 M KOH, as shown in Figure 7a. At a current density of 10 mA/cm^2^, the overpotential of NiCoW is 300 mV, that of NiCo-2 is 340 mV, and that of NiCo-1 is 320 mV. The overpotential of NiCoW is lower than that of the two binary metals. This result indicates that NiCoW has a lower overpotential at the same current density, which is due to the enhanced synergistic effect of the elements with the addition of W. From the Tafel slopes in Figure 7b, the Tafel slope of NiCoW is 90.3 mV/dec, which is smaller compared to 117.1 mV/dec for NiCo-2 and 105.4 mV/dec for NiCo-1, indicating that NiCoW has faster reaction kinetics. The impedance spectra can demonstrate the resistance in the electron transfer process during OER, elucidating the relationship between the catalytic activity of the material and its electrical conductivity. After equivalent circuit simulation, as shown in Figure 7c, the impedance of the NiCoW catalyst with W doping is 2.50 Ω, compared to 2.73 Ω for NiCo-2 and 4.86 Ω for NiCo-1 during OER. This indicates that NiCoW has lower mass transfer resistance compared to the two NiCo catalysts, suggesting that the incorporation of W effectively reduces the charge transfer resistance [44,45]. The histogram in Figure 7d compares the overpotentials of NiCoW, NiCo-2, and NiCo-1 at current densities of 10 mA/cm^2^ and 100 mA/cm^2^. Figure 8a–c shows the CV curves obtained from testing the samples in KOH solution. The number of active sites on the catalyst can be characterized by ECSA, which is proportional to the double-layer capacitance (C_dl_) measured by CV. As shown in Figure 8d, the C_dl_ values for NiCoW, NiCo-2, and NiCo-1 are 3.43, 0.91, and 1.25 mF cm⁻^2^, respectively. The higher C_dl_ value of NiCoW compared to the other two catalysts indicates that the doping of W provides more active sites for the OER reaction, thereby accelerating the adsorption of intermediates and promoting oxygen evolution, ultimately enhancing the OER performance.

### 3.3. UOR Performance

**Figure 9:**
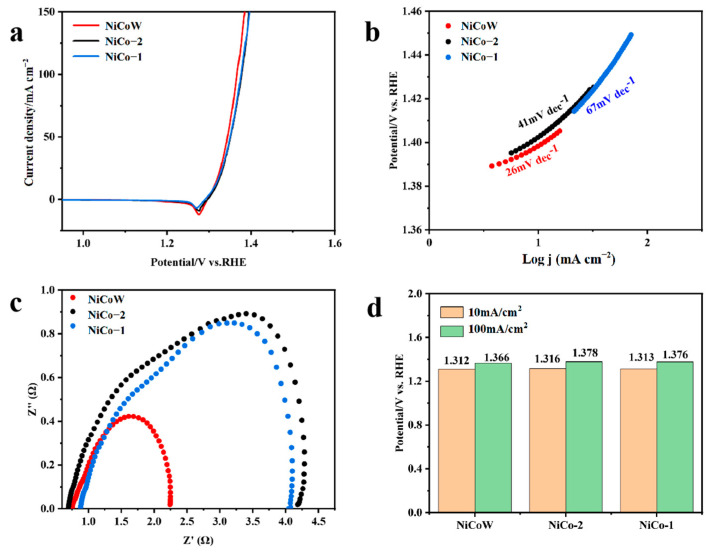
(**a**) Polarization curves of NiCo-1, NiCo-2, and NiCoW catalysts for 1 M KOH electrolyte with and without 0.5 M urea. (**b**) Tafel slopes of NiCo-1, NiCo-2, and NiCoW catalysts. (**c**) Nyquist plots of NiCo-1, NiCo-2, and NiCoW catalysts. (**d**) The histogram of potentials at 10 and 100 mA cm^−2^.

**Figure 10:**
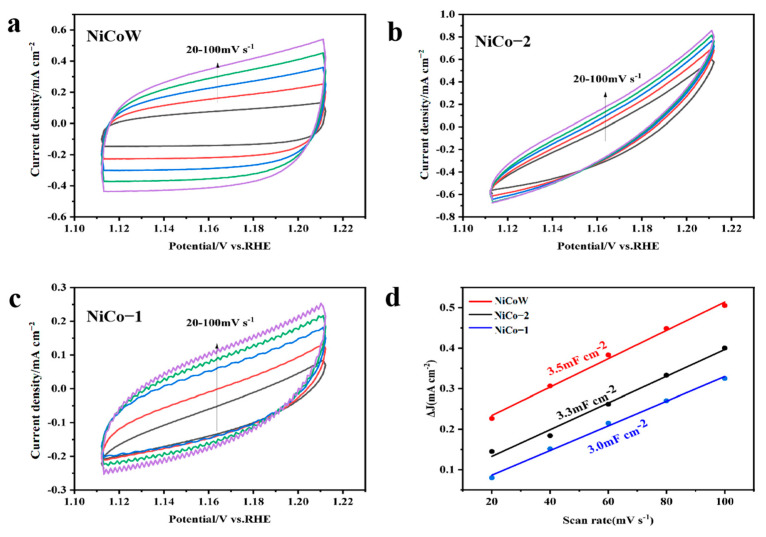
(**a**–**c**) Typical CV curves of 1.0 M KOH + 0.5 M urea, scan rates range from 20 to 100 mV s^−1^. (**d**) C_dl_ of NiCoW, NiCo-1, and NiCo-2 catalysts. The different colors of the curves in the figure mean that we performed the CV cycles at different sweep speeds.

The UOR performance of the samples can be evaluated in a 1 M KOH alkaline solution with 0.5 M urea. As shown in Figure 9a, at a current density of 10 mA/cm^2^, the potentials of NiCoW, NiCo-2, and NiCo-1 are 1.312 V, 1.316 V, and 1.313 V, respectively. At a current density of 100 mA/cm^2^, the potentials are 1.366 V, 1.378 V, and 1.376 V, respectively. It can be seen that NiCoW has lower potentials than the two NiCo catalysts, indicating the best catalytic activity. Figure 9b shows the Tafel slopes of NiCoW and two NiCo catalysts during the UOR process. The Tafel slope of NiCoW is 26 mV/dec, lower than 41 mV/dec for NiCo-2 and 67 mV/dec for NiCo-1. The smallest Tafel slope of NiCoW indicates faster reaction kinetics. Electrochemical impedance spectroscopy was used to evaluate the charge transfer resistance between the electrolyte and catalyst during the UOR process. Figure 9c shows the resistance values obtained after equivalent circuit simulation during UOR. It can be observed that NiCoW has the lowest charge transfer resistance (Rct) among the three samples, indicating faster electron transfer rates, which is advantageous for the UOR reaction. The histogram in Figure 9d compares the overpotentials of NiCoW, NiCo-2, and NiCo-1 at current densities of 10 mA/cm^2^ and 100 mA/cm^2^. Figure 10a–c shows the CV curves obtained from testing the samples in KOH and 0.5 M urea solution. Figure 10d shows the current–voltage variations of the three samples during UOR at scan rates of 20–100 mV/s. After fitting the calculations, the C_dl_ values for these materials can be obtained. The C_dl_ values for NiCoW, NiCo1-2, and NiCo-1 are 3.5 mF cm^−2^, 3.3 mF cm^−2^, and 3.0 mF cm^−2^, respectively. The higher C_dl_ value indicates that NiCoW exposes more active sites.

### 3.4. Stability Analysis and Urea-Assisted Overall Water Splitting

**Figure 11:**
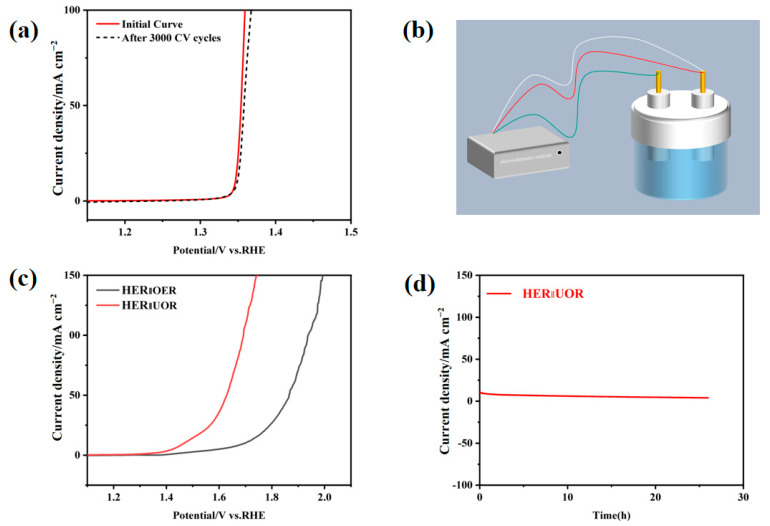
(**a**) LSV curves of NiCoW before and after repeated 3000 CV tests. (**b**) Schematic presentation of overall water-splitting performance. In an electrochemical cell, a two-electrode system is required for measuring complete water solution, in which the green wire connects the working electrode, the anode, and the white and red wire connects the other electrode, the cathode. (**c**) LSV of NiCoW overall water-splitting and (**d**) i-t curve of NiCoW overall water-splitting.

Stability is considered one of the most critical performance metrics for electrocatalysts. The NiCoW catalyst, which exhibits the best overall performance, was subjected to a stability test with 3000 cyclic voltammetry (CV) cycles, as shown in Figure 11a. The linear scan voltammograms reveal that after 3000 CV cycles, the degradation of the NiCoW catalyst is negligible, indicating that the NiCoW catalyst exhibits excellent operational stability [46,47].

To further validate the catalytic performance of the NiCoW catalyst, overall water-splitting tests were conducted using a two-electrode testing system in both urea-containing and urea-free solutions, as shown in Figure 11b. Here, NiCoW was used as both the cathode and anode for the overall water-splitting tests, conducted under both urea-assisted and non-urea-assisted conditions. As shown in Figure 11c, at a current density of 10 mA cm^−2^, the overall water-splitting potential was 1.69 V. After the addition of a certain amount of urea, the potential decreased to 1.46 V due to the enhanced anodic reaction, indicating a significant improvement in the overall water-splitting performance of the NiCoW catalyst. An electrochemical workstation performed a 25 h i-t test at a voltage corresponding to 10 mA cm^−2^ to characterize the stability of the catalyst, as shown in Figure 11d. After 25 h of testing, the potential change was negligible, indicating that the NiCoW catalyst exhibits good electrocatalytic stability.

### 3.5. OER and UOR Mechanism

**Figure 12:**
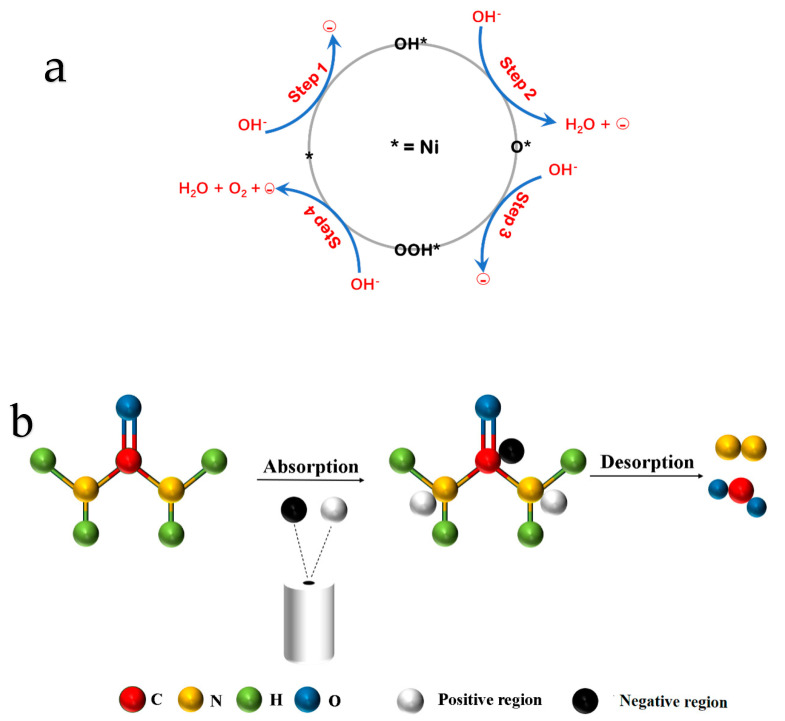
Mechanisms corresponding to OER (**a**) and UOR (**b**) processes.

The OER catalytic process involves a sequence of four consecutive electron and proton transfer steps, as illustrated in Figure 12a. Firstly, hydroxide ions are adsorbed onto the catalytic site (*) to form OH. Then, OH* interacts with OH– to deprotonate and form O*, and O* adsorbs OH– to form OOH*. Finally, OOH* adsorbs OH– to produce oxygen gas and free up the catalytic site, completing the cycle.

The UOR is a multi-step electron–proton coupling process. Although the details of each step in the UOR are not yet fully understood, it is known to involve a six-electron transfer process [48,49]. Figure 12b illustrates the mechanism, where urea molecules react with the active sites of the catalyst to produce nitrogen gas, carbon dioxide, and water. The theoretical potential at the anode is −0.46 V (vs. SHE), and at the cathode, it is −0.83 V (vs. SHE). Therefore, a voltage of 0.37 V is required to sustain the entire reaction process [50,51]. Such a low theoretical potential not only enhances the reaction rate but also reduces energy consumption in the hydrogen production process, achieving a win–win situation.

## 4. Conclusions

In summary, we can prepare a three-dimensional nanonetwork structure of a NiCoW metallic compound using an ultrasonic-assisted NaBH_4_ reduction method. The three-dimensional nanonetwork structure features interconnected metal ligaments and intricate nanopores, resulting in a higher specific surface area that exposes more active sites. This promotes reactant transport and electron conduction, accelerating the reaction. Additionally, the incorporation of metal W enhances the synergistic effect between elements, further improving the catalytic performance of the material. Characterization and performance tests reveal that the NiCoW catalyst exhibits excellent electrocatalytic performance and long-term stability. At a current density of 10 mA cm^−2^, the NiCoW sample has a potential of 1.53 V during the OER process and 1.31 V during the UOR process. This indicates that, under the influence of electronic and morphological modulation, the catalyst demonstrates excellent catalytic performance. Similarly, when the NiCoW catalyst is used as both the cathode and anode for overall water splitting, the potential is 1.69 V. When urea is added to the electrolyte, the potential decreases by 0.22 V at a current density of 10 mA cm^−2^. Furthermore, during long-term cyclic stability testing, the NiCoW catalyst also demonstrates excellent stability and durability. It is evident that NiCoW is a highly promising catalytic material. This study also provides a new approach for developing new transition metal-based UOR electrocatalysts with good electrocatalytic activity and stability.

## Figures and Tables

**Table 1:** Table of electrocatalytic performance parameters of noble metals.

**HER**	**Electrocatalysts**	**Electrolytes**	**Overpotential at 10 mA cm** **^−2^ (mV)**	**Overpotential at 50 mA cm** **^−2^ (mV)**
	**Pt/C**	**1 M KOH**	**40**	**74**
	**RuO_2_**	**1 M KOH**	**89**	**150**
UOR	Electrocatalysts	Electrolytes	Potential at 10 mA cm^−2^ (V)	Potential at 50 mA cm^−2^ (V)
	Pt/C	1 M KOH + 0.5 M urea	1.37	1.44
	RuO_2_	1 M KOH + 0.5 M urea	1.41	1.43

**Table 2:** The relative contents of elements.

	Name	Atomic%
NiCo-2	Ni2p	8.48
	Co2p	25.55
	C1s	18.41
	O1s	47.57
NiCoW	Ni2p	7.56
	Co2p	13.10
	W4f	0.85
	C1s	12.17
	O1s	66.32

**Table 3:** Structural parameters and BET-specific surface areas of samples.

Catalyst	BJH Pore Size (nm)	BJH Pore Volume (cm^3^/g)	BET (m^2^/g)
NiCo-2	89.0	0.249	145.5
NiCoW	17.1	0.395	165.1

## Data Availability

The original contributions presented in the study are included in the article, further inquiries can be directed to the corresponding author.
